# Female Sex Workers’ Experiences of Violence and Substance Use on the Haitian, Dominican Republic Border

**DOI:** 10.5334/aogh.2889

**Published:** 2020-08-20

**Authors:** Kristine R. Hearld, Henna Budhwani, Macarena Martínez-Órdenes, Amber Altaf, Julia Hasbun, John Waters

**Affiliations:** 1 University of Alabama at Birmingham, US; 2 Universidad San Sebastian, CL; 3 Princeton University, US; 4 Caribbean Vulnerable Communities Coalition, JM

## Abstract

**Background:**

Female sex workers (FSW) are socially and economically marginalized, and this vulnerability can be exacerbated when they hold the intersectional identity of also being an immigrant, such as in the case of Haitian FSWs in the Dominican Republic.

**Objective:**

Considering that half of migratory young women and girls relocating across the Latin American and Caribbean region do so without their families, increasing the likelihood of experiencing abuses, our primary objective was to test the hypothesis that Haitian FSWs in the Dominican Republic have higher odds of being physically abused by sexual partners compared to Haitian FSWs in Haiti.

**Methods:**

We conducted bivariate analyses and multivariate analyses on 2014 Hispaniola Sex Workers Study (N = 232).

**Findings:**

Approximately 80% of Haitian FSWs in the Dominican Republic reported experiencing violence by a regular partner (80.3%), compared with 60.0% of Haitian FSWs in Haiti (χ^2^ = 11.34, p < 0.001). Controlling for socio-demographics, substance use, childhood abuse, and sexual behaviors, Haitian FSWs in Haiti maintained lower odds of experiencing violence by a regular partner (OR:0.37, p < 0.01) and higher odds of experiencing violence from a coworker (OR:6.38, p < 0.001) compared to FSWs in the Dominican Republic. Using sex to avoid arrest is associated with higher odds of experiencing violence by a client and violence by a coworker (OR:2.18, p < 0.05; OR:3.74, p < 0.001; respectively). Accepting payment in the form of drugs/alcohol is associated with higher odds of experiencing physical violence by a regular partner but lower odds of experiencing violence by a client (OR:3.99, p < 0.05; OR:0.43, p < 0.05; respectively).

**Conclusions:**

Assuming health is a human right, then practitioners and scholars must actively collaborate to fortify vulnerable populations against injurious structural and sociocultural forces examining the intersectionality and compound effects of multiple stigmatized identities, in this study being an FSW and an immigrant, that moderate the potential positive effects of public health interventions.

## Introduction

Female sex workers (FSW) are socially and economically marginalized [1,2,3]; their vulnerability is exacerbated when FSWs are also immigrants, as is the case of many Haitian FSWs who live and work on the Dominican Republic side of the Dominico-Haitian border [2,4]. In fact, most of the bi-national migration across the entire Latin American and Caribbean (LAC) region occurs between Haiti and the Dominican Republic, with about half of migratory young women and girls relocating without their families, increasing the likelihood that they may experience a range of abuses leading to substance use for coping and survival [1,2,3]. Additionally, Haitian FSWs in the Dominican Republic may have insufficient language proficiency (speaking French Creole fluently but unable to communicate in Spanish, the predominant language spoken in the Dominican Republic), and the physical locations where they routinely engage in sex work (e.g., on the street, in semi-public places, etc.) increase the likelihood of experiencing stigmatizing attitudes and abusive behaviors [2,4,5,6]. Due to the dearth of border studies conducted with FSWs, we evaluated data from the 2014 Hispaniola Sex Workers Study to characterize Haitian FSW respondents’ experiences working on both sides of the Dominico-Haitian border. Informed by findings from global studies with FSWs and Goffman’s conception of stigma wherein the “other” (in this case, Haitian FSWs in the Dominican Republic) may be societally devalued leading to increased negative exposures and outcomes, we hypothesize that Haitian FSWs in the Dominican Republic will have higher odds of being physically abused by sexual partners as compared to Haitian FSWs in Haiti. We also hypothesize that these experiences of violence and abuse will be associated with substance use, a well-documented method to cope with trauma and distress [7,8,9].

Addressing violence against FSWs is a global public health priority [10]. FSWs routinely encounter both intimate partner violence and gender-based violence [10,11,12,13]. In the context of FSWs’ lives, violence is not only initiated by clients and non-commercial partners, but other key actors in the sex work industry also commit such abusive acts, namely bar owners, police, and even other FSW [12]. For FSWs working at geographic borders, financial need is often a major motivation to initiate sex work; in fact, many women that engage in commercial sex work are mothers with limited options for work, making the sex work industry a pathway to financial independence [12,13,14,15]. However, to continue in sex work, these women may partake in alcohol and drug use to initiate encounters, as well as to help them cope with their experiences.

The World Health Organization reports that Haitians consume an average of 6.4 liters of alcohol per capita annually while those in the Dominican Republic consume an average of 6.9 liters of alcohol per capita annually; 10% of the Haitian population and nearly 30% (29.4%) of Dominican Republic residents engage in heavy episodic drinking [16,17]. Both nations report notable years of life lost related to alcohol use; two years for Haiti and three years for the Dominican Republic [18]. Because sex work is highly stigmatized, FSWs employ strategies to cope, with substance use (both legal and illegal) being the leading mechanism [19]. This association between physical abuse and substance use to cope is not solely applicable to FSWs. A series of recent studies with transgender women in the Dominican Republic found that substance users experienced higher rates of trauma, torture, and sexual abuse as compared to those who abstained [20,21]. When this trend – traumatic experiences being associated with substance use to cope – occurs within cultural contexts that already embrace high levels of alcohol consumption, victims of violence may move from substance use to substance abuse to addiction, inflicting further harm on themselves.

Relative to those in other occupations, sex workers have a drug use incidence rate that is greater. However, it is important to note that sex work alone may not result in heightened substance use. FSWs with certain attributes, namely being in poverty, being previously abused, and having limited educational access, predisposes young women to both substance use and sex work. In fact, a study on the determinants of HIV among FSWs in Kenya found that 80% of respondents exchanged sex while intoxicated [22]. Also, a study of nightclub-based FSWs reported higher levels of binge drinking compared to FSWs in bars or lodges [22,23]. Thus, it is important to take note of multiple factors that can influence substance use among FSWs rather than only focuses on the occupation itself. Other studies have confirmed that sex workers used alcohol and drugs as a way to “relieve stress,” “pass time,” and/or “instill courage” before having sex with a client [24,25,26]. That being said, there appears to be a trend across global studies of sex workers that indicates FSWs may use alcohol to facilitate engagement in sex work [27]. Considering these findings, we evaluated the rates of three sources of violence experienced by FSWs working in the Dominican Republic and Haiti (N = 232): physical violence by a regular partner, physical violence by a client, and physical violence by a coworker.

## Methods

### Study Design and Participants

Haitian FSWs were recruited between February 2014 to June 2014 for the Hispaniola Sex Workers Study. Data were collected by the Caribbean Vulnerable Communities Coalition (CVC). Associated STI screening and treatment were provided by El Centro de Promocion y Solidaridad Humana (CEPROSH), a local non-governmental organization (NGO) that provides sexual and reproductive health services along the border. There were three criteria for participant inclusion in the study: 1) Having exchanged goods or money for sexual acts in the past year, 2) Being at least eighteen years old, and 3) Identifying as a Haitian national. Venue-based sampling was employed to identify days and times that sex workers gather at high-traffic venues, constructing a sampling frame of venue, day, time units (VDT). Recruiters then randomly selected and visited these VDTs (primary sampling unit) and systematically collected survey responses from eligible participants. Surveys were available in Haitian Creole and Spanish and administered in the language of preference of the Haitian women surveyed. A participant incentive in the form of a travel stipend to cover transportation costs, as well as a referral card for STI screening and treatment, were given to study participants.

El Consejo Nacional de Bioética en Salud (CONABIOS) in the Dominican Republic provided ethical approval for this study. Ethical approval for the secondary analysis of these de-identified data was obtained from the University of Alabama at Birmingham’s (UAB) Institutional Review Board (#IRB300001560, FWA00005960).

### Measures, Violence Outcomes

The dependent variables were three types of violence: physical violence by a regular partner, physical violence by a client, and physical violence by a coworker. Respondents were asked the following questions: “Have you ever been a victim of physical violence by your partner,” “Have you ever been a victim of physical violence by your client,” and “Have you ever been a victim of physical violence by a coworker.” For all three questions, FSWs who responded “yes” were assigned as 1 and “no” as 0.

### Measures, Control Variables

Three socio-demographic independent variables were included: age, educational attainment, and country of work. Age was measured as a continuous variable in years. Education was measured as a categorical variable consisting of no education (referent), primary school or less, and secondary school. The country was a country where participants currently worked; response options were Haiti or the Dominican Republic.

Alcohol use variables were dichotomous. Respondents indicated if they ever accepted drugs or alcohol as payment for services, and if they used alcohol to facilitate sex work (1 = yes/sometimes; 0 = no). Similarly, childhood abuse was measured with dichotomous variables for physical and verbal abuse. Respondents reporting that her mother, father, or surrogate had engaged in that type of abuse (physical or verbal) were coded as 1; otherwise were coded as 0. Sexual behaviors were measured with two continuous and one dichotomous variable: 1) Age of first sexual experience with penetration; 2) Age of first engagement in sex work; and 3) Used sex to avoid arrest (1 = yes; 0 = no).

### Analytic Strategy

Univariate and bivariate analyses were conducted (N = 232). Multivariate analyses identified correlates of abuse. All models were estimated with Stata 15.0.

## Results

Sixty-eight observations were dropped from the analysis due to missing data on key variables; our final sample included two-hundred and thirty-two Haitian FSWs (N = 232). Ninety (38.8%) self-identified as working on the Haitian side of the border and 142 (61.2%) as working on the Dominican Republic side of the border. Table 1 presents the sample characteristics. The average age of Haitian FSWs on the Dominican Republic side was 27.1 years and 28.8 years for FSWs on the Haiti side (t = –4.33, p < 0.001). On average, educational attainment was similar (low) among respondents who worked on both sides of the border, with over 91% of respondents having attained a primary or lower level of education (91.1%).

**Table 1:** Characteristics of female sex workers on both sides of the border (N = 232).

	Dominican N = 142	Haitian N = 90	Total N = 232	χ^2/t^

**Violence and Abuse**				
Physical violence by regular partner	114 (80.28%)	54 (60.00%)	168 (72.41%)	**χ^2^= 11.34** **p < 0.001**
Physical violence by client	86 (60.56%)	53 (58.89%)	137 (59.91%)	χ^2^ = 0.06 p = 0.80
Physical violence by coworker	85 (59.86%)	74 (82.22%)	159 (68.53%)	**χ^2^ = 12.78** **p < 0.001**
**Socio-demographics**				
Age (Mean/Standard Deviation)	27.12 (2.77)	28.81 (3.10)	27.31 (2.85)	**t = –4.33** **p < 0.001**
Education				
Primary school or less	134 (94.37%)	82 (91.11%)	216 (93.10%)	χ^2^ = 0.91
≥ Some secondary school	8 (5.63%)	8 (8.89%)	16 (6.90%)	p = 0.34
**Substance Use**				
Paid by clients in drugs or alcohol	35 (24.65%)	12 (13.33%)	47 (20.26%)	**χ^2^ = 4.37** **p < 0.05**
Use of alcohol to facilitate sex work	119 (83.80%)	72 (80.00%)	191 (82.33%)	χ^2^ = 0.55 p = 0.46
**History of Childhood Abuse**				
Physical abuse	95 (66.90%)	37 (41.11%)	132 (56.90%)	**χ^2^ = 14.48** **p < 0.001**
Verbal abuse	123 (86.62%)	67 (74.44%)	190 (81.90%)	**χ^2^ = 5.51** **p < 0.05**
**Sex Behavior**				
Age of first engagement in sex with penetration	14.01 (9.18)	15.56 (3.76)	14.36 (7.02)	t = –1.52 p = 0.13
Age of first engagement in sex work	15.76 (8.06)	18.13 (3.40)	16.39 (6.20)	**t = -2.65** **p < 0.01**
Avoid arrest through sex	89 (62.68%)	44 (48.89%)	133 (57.32%)	**χ^2^ = 4.28** **p < 0.05**

Abuses experienced on both sides of the border were high, although generally higher among Haitian FSWs working on the Dominican Republic side of the border. Approximately 80% of Haitian FSWs on the Dominican Republic side of the border reported experiencing physical violence by a regular partner (80.3%), compared with 60.0% of the Haitian FSWs on the Haitian side of the border (χ^2^ = 11.34, p < 0.001). The one area of abuse that was higher on the Haitian side of the border was physical violence by a coworker. Over 80% of FSWs working on the Haitian side of the border experienced physical violence by a coworker (82.2%), compared to 59.9% of FSWs on the Dominican Republic side of the border (χ^2^ = 12.78, p < 0.001). Two-thirds of Haitian FSWs working on the Dominican Republic side reported experiencing physical abuse from a parent (66.9%), compared to 44.1% of the Haitian FSWs working on the Haitian side (χ^2^ = 14.48, p < 0.001). Similarly, 86.6% of Haitian FSWs on the Dominican Republic side indicated that they had experienced verbal abuse from a parent, compared to 74.4% of the Haitian FSWs on the Haitian side (χ^2^ = 5.51, p < 0.001).

Age of first engagement in sex work was earlier for Haitian FSWs working on the Dominican Republic side of the border; mean age of 15.8 years in the Dominican Republic compared to 18.1 years in Haiti (t = –2.65, p < 0.01). Two-thirds of Haitian FSWs on the Dominican Republic side of the border indicated that they had sex with a law enforcement agent to avoid arrest or deportation (62.7%), compared to 48.8% of the Haitian FSWs on the Haitian side of the border (χ^2^ = 4.28, p < 0.05). One-quarter of the Haitian FSWs on the Dominican Republic side of the border reported being paid by clients in drugs or alcohol (24.7%), compared to 13.3% of the FSWs on the Haitian side of the border (χ^2^ = 4.37, p < 0.05).

We present multivariate logistic regression results for three outcomes: Physical violence by a partner, physical violence by a client, and physical violence by a coworker, in Table 2. Controlling for socio-demographics, substance use, history of childhood abuse, and sexual behaviors, Haitian FSWs in Haiti maintained lower odds of experiencing violence by a regular partner and discrimination (OR:0.37, p < 0.01) and higher odds of experiencing violence from a coworker (OR:6.38, p < 0.001) compared to FSWs in the Dominican Republic.

**Table 2:** Correlates of physical violence among female sex workers with three partner types: regular partner, client, a coworker (N = 232).

	Physical violence regular partner OR 95% CI	Physical violence client	Physical violence coworker

**Socio-demographics**			
Nationality Dominican Haitian	Referent **0.365 ** 0.177–0.754**	Referent 1.524 0.725–3.205	Referent **6.383 ** 2.532–16.091***
Age	**1.192** 1.040–1.366*	1.040 0.930–1.158	1.038 0.930–1.158
Education Primary education or less Secondary education or higher	**7.726** 2.257–26.450** Referent	0.917 0.221–3.814 Referent	0.917 0.221–3.914 Referent
**Substance Use**			
Use of alcohol to facilitate sex work	1.263 0.541–2.949	**6.400** 2.602–15.743***	**6.878** 2.622–18.038***
Paid by clients in drugs or alcohol	**3.992** 1.113–14.317*	**0.429** 0.198–0.930*	2.159 0.875–5.326
**History of Childhood Abuse**			
Physical abuse	1.198 0.570–2.518	**2.819** 1.400–5.676**	2.058 0.951–4.455
Verbal abuse	0.566 0.210–1.524	0.446 0.176–1.130	**0.027** 0.003–0.2494***
**Sex Behavior**			
Age of first engagement in sex with penetration	0.960 0.918–1.002	0.920 0.824–1.027	1.053 0.928–1.196
Age of first engagement in sex work	0.959 0.915–1.004	1.002 0.946–1.060	0.983 0.930–1.038
Avoid arrest through sex	**2.177** 1.061–4.466*	**3.737** 1.951–7.160***	1.447 0.715–2.928

Increases in age were associated with higher odds of physical violence by a regular partner (OR:1.19, p < 0.05). Having a primary or lower level of educational attainment was associated with higher odds of physical violence by a regular partner (OR:7.73, p < 0.01). Using alcohol to facilitate sex work was associated with higher odds of experiencing violence by a client and a coworker (OR:6.40, p < 0.001; OR:6.88, p < 0.001; respectively). A history of parental verbal abuse is associated with lower odds of experiencing violence by a coworker (OR:0.03, p < 0.001). Using sex to avoid arrest is associated with higher odds of experiencing violence by a client and violence by a coworker (OR:2.18, p < 0.05; OR:3.74, p < 0.001; respectively). Accepting payment in the form of drugs or alcohol for sex work is associated with higher odds of experiencing physical violence by a regular partner but lower odds of experiencing violence by a client (OR:3.99, p < 0.05; OR:0.43, p < 0.05; respectively).

## Discussion

We found support for our hypothesis that Haitian FSWs in the Dominican Republic would have higher odds of being physically abused by sexual partners, as compared to Haitian FSWs in Haiti. We also found that exposure to abuse was associated with substance use. The self-reported rate of experiencing physical violence by a regular partner, as well as having been discriminated against by law enforcement, was higher in Haitian FSWs on the Dominican Republic side of the border as compared to their peers on the Haitian sides of the border. Additionally, FSWs on the Dominican Republic side of the border reported higher levels of physical violence by coworkers. On the Dominican Republic side of the border, 62.7% of respondents indicated that they had sex with a policeman or law enforcement agent (coercion) to avoid arrest or deportation. And, these negative experiences were, indeed, associated with using alcohol to facilitate sex work for accepted drugs and alcohol as payment for sex work.

Although outside the primary scope of our study, we also found alarming rates of childhood abuses; most FSWs on the Dominican Republic side of the border experienced verbal abuse and/or physical abuse during childhood; about two-thirds of FSWs on the Haiti side of the border experienced verbal abuse in childhood with less than half having to experience physical abuse. Prior research has shown that these early negative experiences increase the likelihood that children will grow into adults who are more likely to engage in high-risk health behaviors, such as drug and alcohol abuse, and sex work [28]. These findings are important for applied public health practice and policies related to sex work, but what is also theoretically valuable from this study is the role of intersectionality. It is well documented that sex work is stigmatized; we also know that being an immigrant can be stigmatizing [29,30] but what we see in this study, although we cannot explicitly test of it, is the effects of intersectional stigmatized identities namely being a Haitian immigrant in the Dominican Republic who is engaged in sex work. More research needs to be conducted to better understand how to address this intersectionality to bolster these FSWs’ internal psychological processes while also reduces exposures to violence.

## Limitations

When extending or applying our findings, limitations should be considered. First, self-report and recall bias is likely. For example, although we parse outcomes by partner type, respondents may not remember exactly which type of partner, particularly between paying clients and non-commercial partners. Partners may also exist in multiple categories; a client may become a non-commercial, coercive partner on a different day. Second, some terms were not explained within the delivery of the questionnaire. For instance, we did not explain to respondents what we felt constituted a ‘client;’ thus, some responses to some questions may have varied slightly between respondents. Third, our data is not representative of all FSWs, and as part of our inclusion criteria, we excluded sex workers who seek seasonal work and may arrive in the Dominican Republic and Haiti from other Caribbean nations (e.g., Guyana, Dominica, etc.). Lastly, since our data is cross-sectional, our findings are not generalizable. We cannot infer causal inferences. Even with these limitations, our dataset is one of the few repositories that contains self-reported information from FSWs on a geographic border.

## Implications

Our study lays the foundation for future research and improved care delivery for sex workers on geographic borders and, more broadly speaking – in resource-constrained settings. These improvements could include tailored health policy addressing risk reduction in stigmatized, vulnerable, and hard-to-reach populations.

## Conclusion

If health is truly a human right, as the World Health Organization suggests, then practitioners and scholars must actively collaborate to fortify vulnerable populations against injurious structural and sociocultural forces (that lead to high-risk behaviors) through the implementation of tailored policies and practice-interventions that include universal protections.
